# Case Report: Ramipril and microscopic colitis; a necessary tool of cardiologists can rarely be devastating for patients

**DOI:** 10.12688/f1000research.25552.1

**Published:** 2020-09-09

**Authors:** Saad Hasan, Haseeb Ur Rahman, Stephen Hutchison

**Affiliations:** 1 Cardiology Department, Nevill Hall Hospital, Abergavenny, Monmouthshire, NP7 7EG, UK

**Keywords:** Ramipril, microscopic colitis, diarrhoea, bowel incontinence

## Abstract

Angiotensin converting enzyme inhibitors could lead to severe diarrhoea related to microscopic colitis. Few of such cases have been reported before and this serious problem, from a widely used class of drugs in hypertension and heart failure, needs to be more recognised. We describe the case of collagenous colitis related to ramipril use in the following case report.

A 74-year-old farmer who had a history of triple vessel coronary artery disease was admitted to district general hospital with non-ST elevation myocardial infarction. He had known alcohol-related chronic pancreatitis with chronic diarrhoea as a complication, which was managed with pancreatic enzyme replacement therapy. However, he developed severe worsening of diarrhoea causing bowel incontinence and nocturnal symptoms during his admission to hospital. The explosive and watery nature of diarrhoea with urgency was so troublesome that it delayed coronary revascularisation and lead him to have significant psychological distress and low mood while nocturnal bowel motions meant he was unable to sleep. He was compliant with his pancreatic enzyme replacement therapy during this period. Infective causes were ruled out by stool microbiology examination and coeliac disease by oesophagogastroscopy and biopsy. It was noticed that he was recently prescribed ramipril that was later stopped as a possible diarrhoea trigger. Diarrhoea started settling immediately and resolved to his baseline within a week. A colonoscopy was performed in the meantime and biopsies demonstrated microscopic colitis (MC). He did not tolerate budesonide well so was stopped. However, a follow-up colonoscopy with biopsy in two months showed resolution of MC.

## Introduction

Angiotensin converting enzyme inhibitors (ACE-I) are widely used in the treatment of hypertension and heart failure and considered as a safe option. They are usually well tolerated in up to 90% of patients ^1^ while diarrhoea is noticed in up to 8% ^2^. We report a case of severe diarrhoea associated with ramipril use.

## Case report

A 74-year-old man, farmer by profession, admitted from the A&E department where he presented with non-specific upper abdominal pain involving epigastrium and right upper quadrant. He had been drinking excessive alcohol for the previous three days. There was a previous history of alcohol excess, but he had been abstinent for ten years. The patient’s past medical history also included triple vessel coronary artery disease, for which he was awaiting revascularisation planned in three weeks time. He also had alcohol-related liver cirrhosis, chronic pancreatitis, diverticular disease, hypothyroidism and asthma. His regular medications included bisoprolol, lansoprazole, clopidogrel, isosorbide mononitrate, levothyroxine, atorvastatin, spironolactone, citalopram, amlodipine, thiamine and pancreatic enzyme replacement. He lived with his wife and was independent with activities of daily living.

On examination, the patient’s blood pressure was 79/51 mmHg, pulse 84/min, respiratory rate 17 and he was afebrile. Abdominal and chest examinations were within normal limits. Electrocardiography showed normal sinus rhythm with heart rate of 85 per minute. No ischemic changes were noticed. Computed tomography (CT) scan of abdomen and pelvis did not conclude acute pancreatitis or bowel obstruction but already known diverticular disease.

Blood tests revealed raised high sensitivity troponin of 422 nanogram/litre (ng/L) with subsequent values at 3 hours and 6 hours being 403 ng/l and 385 ng/L respectively (normal range 0 – 34 ng/L). Serum amylase was normal. He was treated as non-ST elevation myocardial infarction with standard therapy. Ramipril was also commenced at 2.5 mg once daily dose. He was then planned for inpatient revascularisation.

In about 7 to 10 days of starting ramipril, the patient developed severe exacerbation of his ongoing chronic diarrhoea when he started opening his bowels fifteen to twenty times from about four to five times a day. The bowel motions were watery and explosive in nature with associated severe urgency leading to bowel incontinence episodes, which occurred on a daily basis. As a part of work up of this diarrhoea, stool samples to look for *Clostridium difficile* toxin, salmonella, shigella, Campylobacter, *Escherichia coli* 0157, ova, cysts, parasites and Cryptosporidium were sent, which all came back as negative. Faecal elastase could not be tested because of excessively watery stool, as artificially low elastase levels are expected in such samples. Oesophagogastroscopy with duodenal biopsies did not explain the cause of diarrhoea.

During the period in which diarrhoea was being investigated, which had also delayed the planned cardiac intervention, the patient continued to suffer with severe debilitating symptoms. He was unable to get good sleep at night due to the sudden urges to open bowels, which often lead to incontinence. He would have to change his trousers about two to three times on average every night due to this. It also understandably resulted in severe psychological distress and low mood. This was a cause of concern amongst medical team looking after him and a sense of urgent need to get to the bottom of this issue surfaced.

The patient was then planned for repeat CT scan of the abdomen and pelvis after specialist gastroenterology input. It did not show any interval changes in comparison to the previous CT scan performed 4 weeks ago. Comments were made on intra-abdominal vasculature noting heavily calcified abdominal aorta. The origins of the superior mesenteric artery and celiac artery, although calcified, were widely patent.

Drugs were reviewed and lansoprazole withheld to no avail. Afterwards ramipril was stopped approximately a month after it was commenced, which was one of the newly started medications after patient’s admission to the hospital, and this immediately lead to some improvement of symptoms. It was followed up by flexible sigmoidoscopy with biopsies taken from distal sigmoid colon, which showed diffuse increase in chronic inflammatory infiltrate. There was also patchy increase in basement membrane thickening and focal epithelial sloughing as features of collagenous colitis. A trial of budesonide was commenced after the biopsy results came back by gastroenterologist at 3 mg three times daily for two weeks with a plan to reduce it to 3 mg twice daily for two weeks and then 3 mg once daily for two weeks and then to stop. Budesonide caused significant abdominal discomfort and nausea, so it was stopped 15 days later, when the patient was seen in the outpatient clinic by gastroenterologist.

The diarrhoea had continued to settle, and bowel motion frequency had gone back to his pre-hospital admission baseline within one to two weeks of stopping ramipril. He underwent successful staged multi-vessel percutaneous coronary intervention and was discharged home after remaining an inpatient for a total of two months.

The patient was followed up as outpatient after two months with colonoscopy and biopsies taken again which came back as negative. Histology showed normal mucosal crypt architecture and no active inflammation which meant that MC had resolved.

## Discussion

ACE-I have been known to cause diarrhoea owing to different aetiologies, including eosinophilic gastroenteritis ^3^ and bowel angioedema ^4^. MC is less commonly associated with ACE-I ^5^.

MC is a known cause of chronic watery diarrhoea, which can also cause faecal incontinence and abdominal pain. On histology, there is increased intraepithelial lymphocytes in lymphocytic colitis with loss of cell architecture and collagen band in sub- epithelium in collagenous colitis ^6^.

Drugs are often implicated in MC. Common ones are proton pump inhibitors, non-steroidal anti-inflammatory drugs, selective serotonin reuptake inhibitors and beta blockers. ACE-Is also increase the risk of MC ^7^.

Usually conservative measures, such as withdrawing the offending drug, can help resolve mild to moderate MC. To reach a conclusion that a drug is responsible, a thorough history should be taken to establish a relationship between starting the drug and development of symptoms ^6^. Obviously, clinical context needs to be taken on board and more common causes should be ruled out first.

## Conclusion

Although ACE-Is are less commonly associated with MC, they are extensively used and hence can affect a large number of patients. Cardiologists need to be aware of this potential problem.

## Consent

Written informed consent for the publication of the manuscript and any associated images was obtained from the patient.

## Data availability

All data underlying the results are available as part of the article and no additional source data are required.

## References

[1] Amadio P Jr Amadio PB Cummings DM : ACE inhibitors. A safe option for hypertension and congestive heart failure. *Postgrad Med.* 1990;87(1):223–6,231–2,235–43. Review. 10.1080/00325481.1990.11704535 2404266

[2] Mangrella M Motola G Russo F : [Hospital intensive monitoring of adverse reactions of ACE inhibitors]. *Minerva Med.* 1998;89(4):91–7. Italian. 9676174

[3] Barak N Hart J Sitrin MD : Enalapril-induced eosinophilic gastroenteritis. *J Clin Gastroenterol.* 2001;33(2):157–8. 10.1097/00004836-200108000-00014 11468446

[4] Gabriel JG Vedantam V Kapila A : Recognizing a Rare Phenomenon of Angiotensin-Converting Enzyme Inhibitors: Visceral Angioedema Presenting with Chronic Diarrhea-A Case Report. *Perm J.* 2018;22:17–030. 10.7812/TPP/17-030 29236651 PMC5737922

[5] Lucendo AJ : Drug Exposure and the Risk of Microscopic Colitis: A Critical Update. *Drugs R D.* 2017;17(1):79–89. Review. 10.1007/s40268-016-0171-7 28101837 PMC5318339

[6] Park T Cave D Marshall C : Microscopic colitis: A review of etiology, treatment and refractory disease. *World J Gastroenterol.* 2015;21(29):8804–10. Review. 10.3748/wjg.v21.i29.8804 26269669 PMC4528022

[7] Masclee GM Coloma PM Kuipers EJ : Increased risk of microscopic colitis with use of proton pump inhibitors and non-steroidal anti-inflammatory drugs. *Am J Gastroenterol.* 2015;110(5):749–59. Epub 2015 Apr 28. 10.1038/ajg.2015.119 25916221

