# Supplementary material for: Case Report: Ramipril and microscopic colitis; a necessary tool of cardiologists can rarely be devastating for patients
Source: F1000Res. 2020 Sep 9;9:1113. [Version 1] doi: 10.12688/f1000research.25552.1 (PMC8921691; doi:10.12688/f1000research.25552.1)
Supplement: Peer review history

# Peer review: Reviewer response for version 1

**DOI:** 10.5256/f1000research.28199.r125826

**Published:** 2022-03-14

**Authors:** Syed Mujtaba Hasnain Nadir

**Affiliations:** 1 Health Education England, North West Deanery, UK

The case is described in adequate detail and common differentials are excluded by relevant investigations. Furthermore, there is a histology proven diagnosis of Microscopic Colitis.

 As per standard of care, the patient must have been given Aspirin as a treatment for his NSTEMI. Aspirin is a commoner cause of MC. Please specify in your case report whether Aspirin was continued after revascularization? Furthermore, the patient is on a PPI which is also a common cause of MC, was this discontinued?

Are enough details provided of any physical examination and diagnostic tests, treatment given and outcomes?

Yes

Is the case presented with sufficient detail to be useful for other practitioners?

Yes

Is sufficient discussion included of the importance of the findings and their relevance to future understanding of disease processes, diagnosis or treatment?

Yes

Is the background of the case’s history and progression described in sufficient detail?

Yes

Reviewer Expertise:

Gastroenterology

I confirm that I have read this submission and believe that I have an appropriate level of expertise to confirm that it is of an acceptable scientific standard.

# Peer review: Reviewer response for version 1

**DOI:** 10.5256/f1000research.28199.r75085

**Published:** 2020-12-14

**Authors:** Mujeeb Ullah Makki

**Affiliations:** 1 Royal Worcestershire Acute Hospital NHS Trust, Worcestershire, UK

In my opinion, content in this case report is well structured and organized. Appropriate history, examination and investigations were ordered. They have excluded other causes which could contribute to diarrhea in this patient. Author has also provided histological evidence of MC which improved after stopping ACEI. 

 There are few points/editions which I will recommend for this case report. Case report is acceptable for indexing with these minor editions/changes:

- Computed tomography (CT) scan of abdomen and pelvis did not conclude acute pancreatitis or bowel obstruction but already known diverticular disease.(please rephrase sentence).
- Any explanation for complete resolution of chronic diarrhea which was complicated by chronic pancreatitis? Did ACEI settled chronic pancreatitis and lead to complete resolution of chronic diarrhea or patient was still having few episodes of loose stool but had histologically improved MC? 
- The patient was then planned for repeat CT scan of the abdomen and pelvis after specialist gastroenterology input. It did not show any interval changes in comparison to the previous CT scan performed 4 weeks ago(please mention what was CT report 4 weeks ago? Any comments on pancreas?)
- Please recheck references and write according to journal policy. 

Once author has done these changes and discussed these points in his report, it can be accepted for indexing.

Are enough details provided of any physical examination and diagnostic tests, treatment given and outcomes?

Yes

Is the case presented with sufficient detail to be useful for other practitioners?

Yes

Is sufficient discussion included of the importance of the findings and their relevance to future understanding of disease processes, diagnosis or treatment?

Yes

Is the background of the case’s history and progression described in sufficient detail?

Yes

Reviewer Expertise:

Gastroenterology

I confirm that I have read this submission and believe that I have an appropriate level of expertise to confirm that it is of an acceptable scientific standard.
